# Effect of solidification rate on microstructure evolution in dual phase microalloyed steel

**DOI:** 10.1038/srep35715

**Published:** 2016-10-19

**Authors:** A. G. Kostryzhev, C. D. Slater, O. O. Marenych, C. L. Davis

**Affiliations:** 1 School of Mechanical, Materials and Mechatronic Engineering, University of Wollongong, NSW 2500, Australia; 2 Warwick Manufacturing Group, University of Warwick, Coventry, CV4 7AL, United Kingdom

## Abstract

In steels the dependence of ambient temperature microstructure and mechanical properties on solidification rate is not well reported. In this work we investigate the microstructure and hardness evolution for a low C low Mn NbTi-microalloyed steel solidified in the cooling rate range of 1–50 Cs^−1^. The maximum strength was obtained at the intermediate solidification rate of 30 Cs^−1^. This result has been correlated to the microstructure variation with solidification rate.

At present, three major concepts to produce strip steel operate in industry: 1 – casting of a >200 mm thick slab, multi pass rough rolling on a reversing mill, finish rolling on a 5–7 stand continuous rolling mill; 2 – casting of a 50–70 mm thick slab, 0–2 rough rolling passes, finish rolling on a 5–7 stand continuous rolling mill (Compact Strip Production technology); 3 – casting of a <20 mm (on a belt) or <2 mm (on twin rolls) thick strip, followed by direct rolling in 1–2 stands (Strip Casting technology). A decrease in thickness of the cast semi-product allows for substantial decreases in energy consumption and production costs, as a result of reduced hot deformation and potential elimination of the reheating stage. Currently, the CSP technology is used to produce carbon, stainless, silicon rich, high strength microalloyed, and quenched and tempered steels^1^,^2^,^3^,^4^. The capabilities of Strip Casting technology have been demonstrated for commercial manufacturing of carbon, silicon rich, and stainless steels^5^,^6^. Industrial trials to produce Nb, V and Cu microalloyed steels by strip casting have been reported^7^,^8^. Under laboratory conditions, twinning-induced plasticity (TWIP)^9^, dual phase (DP)^10^,^11^ and microalloyed (MA)^12^ steels have been obtained by strip casting. When steel grades are manufactured using thin cast semi-product, attaining the desired microstructure-property relationships remain a challenge, due to two reasons: (i) the reduced amount of hot deformation results in a) sluggish recrystallisation, b) large prior austenite grain size, c) large grain size of low temperature phases, and d) reduced ductility; and (ii) insufficient control of the solidification conditions (in particular, solidification rate) and its effect on chemical homogeneity, solid solute concentrations, particle precipitation and hence the ambient temperature strength and ductility. The significance of these reasons increase with a decrease in thickness of the cast semi-product, which is accompanied by increased solidification rates and decreased amounts of hot deformation. Therefore, an investigation of the effects of solidification rate on the evolution of microstructure and mechanical properties is required.

## Material and Methods

In this paper we present a preliminary study of the room temperature microstructure variation in low C, low Mn, NbTi-microalloyed steel, solidified in the cooling rate range 1–50 Cs^−1^. A steel containing 0.085C, 0.5 Mn, 0.19Si, 0.018Ni, 0.26Cr, 0.11Mo, 0.011Cu, 0.04Al, 0.059Nb, 0.035Ti, 0.002S, 0.01P, and 0.0054N (wt. %) was produced and provided by BlueScope Steel Ltd, Australia. To study solidification of steels a number of techniques have been suggested^13^,^14^,^15^,^16^, full analysis of which is outside of this paper scope. We used a confocal scanning laser microscope because it allowed for *in-situ* observation of the solidification process. Samples of approximately 2 × 2 × 2 mm size were machined out of 10 × 15 × 20 mm blocks cut from a quarter thickness position of a continuously cast slab. Each sample was placed in a sapphire crucible sitting in an alumina sample holder at the focus point of the microscope. Temperature was measured by an R-type thermocouple attached to the base of the crucible. The thermocouple calibration against the melting points of pure Sn, Cu and Fe samples showed a temperature error of ±2 °C. The expected response time for this bare wire, butt welded thermocouple is <50 ms. The chamber was evacuated to −100 mbar pressure before back filling with N6 argon (the argon was passed through 3 scrubbers and particle filters to achieve an oxygen level <2 ppm). This process was repeated three times before each test. The samples were heated to 1600 °C at a rate of 10 °Cs^−1^, held for 120 sec to allow for thorough homogenisation of the chemical composition of the liquid pool, then cooled at 1, 10, 30 and 50 Cs^−1^ to 900 °C, then cooled at 1 °Cs^−1^ to 400 °C and then slowly cooled in air to room temperature. The same cooling rate from 900 to 400 °C (austenite decomposition temperature region) was applied to investigate the effect of only solidification rate on the room temperature microstructure. For optical and scanning electron microscopy (SEM), the solid hemisphere samples of approximately 4 mm diameter were mounted in conductive bakelite, polished and etched with 2% nital using standard metallographic techniques. Foils for transmission electron microscopy (TEM) were prepared using a specially designed cutting procedure, fine polishing on a Leica EM TXP machine and ion milling on a Gatan PIPS machine. Microstructure characterisation was carried out using Leica DMRM optical, JEOL 7001F FEG SEM and JEOL JEM2011 TEM microscopes. Bainite grain size, namely the size of bainite phase regions, was measured as a distance between the ferrite-bainite interfaces across the bainite regions. Bainite plate width was measured as a distance between bainite-bainite interfaces perpendicular to the interfaces. These measurements were performed on all three samples for each of four processing condition using the linear intercept method applied to the stitched optical images showing full sample surfaces. For the analysis of >15 nm particle parameters, 130–750 precipitates were imaged using SEM in each of the two microstructural phases present (ferrite and bainite) for each of the four cooling condition. Precipitate compositions were studied using energy dispersive X-ray spectroscopy (EDS) point analysis on an Aztec 2.0 Oxford SEM EDS system; 50–70 particles were analysed in each phase for each cooling condition. To reduce the beam-sample interaction volume and increase the accuracy of EDS experiments, the SEM microscope was set to operate at 5 kV of accelerating voltage. The matrix spectra were acquired in a reasonable proximity to the studied particles, and the peak height variation between the particle and matrix spectra were used to analyse the particle compositions. A relative number of particles for each chemistry type (to the total number of particles analysed) was used to determine the particle chemistry variation with processing condition. For the analysis of <15 nm particle parameters, 40–360 precipitates were imaged using TEM for selected conditions. The precipitate nature was analysed using selected area diffraction. The foil thickness was measured to be 125 nm; a convergent beam diffraction technique was applied for this measurement^17^. For the determination of dislocation densities, 10–15 representative regions were imaged in two-beam condition near [001] and [011] zone axes in each phase for each of three studied processing conditions. The average microhardness of each phase was measured using 8 indentations made on a Struers Emco-Test DuraScan-70 Vickers hardness tester applying 50 g load. For this load, an indent diagonal was no longer than 20 μm, which is several times shorter than the distance between ferrite-bainite interphases. So small indent sizes assured sampling of each constituent phase.

## Results and Discussion

**Figure 1:**
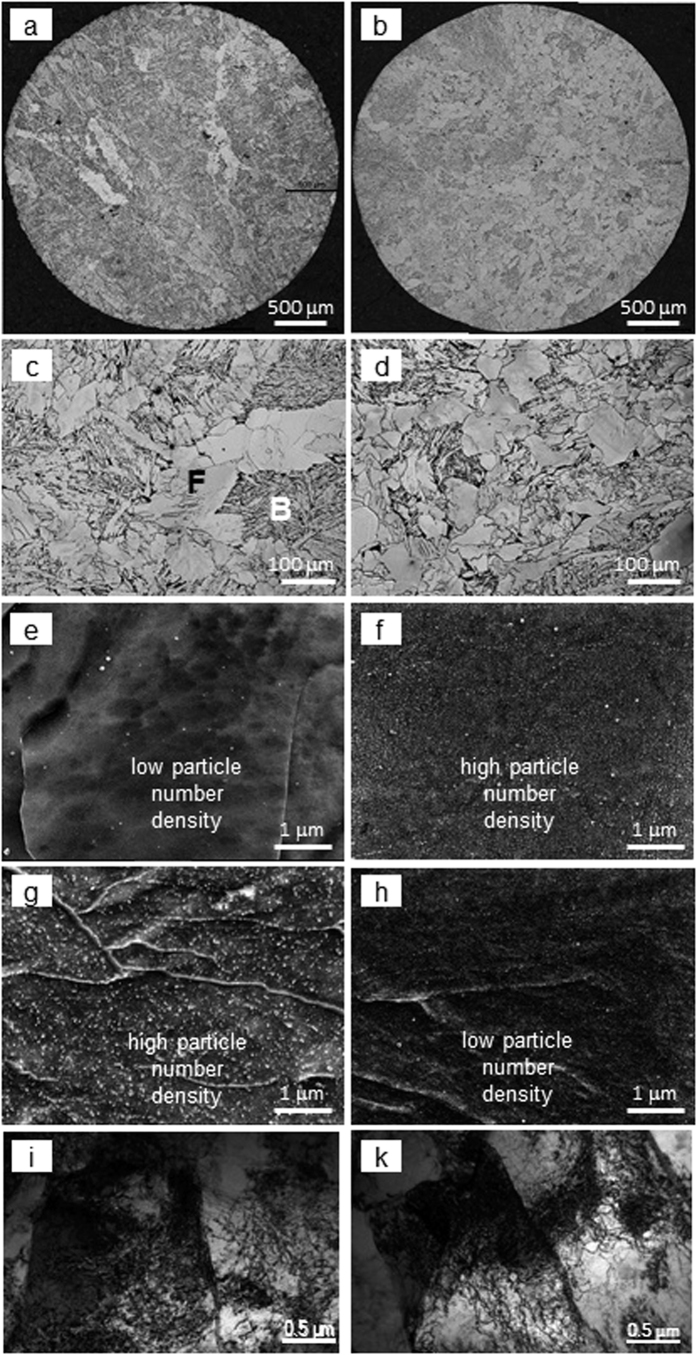
Optical images of the (**a,b**) whole samples used to measure the phase fractions and (**c,d**) selected areas showing ferrite-bainite microstructures (F is ferrite, B is bainite), SEM images of precipitates in (**e**,**f**) ferrite and (**g,h**) bainite, (**i,k**) TEM images of bainite for solidification rates of (**a,c,e,g,i**) 1 Cs^−1^ and (**b,d,f,h,k**) 30 Cs^−1^.

**Figure 2:**
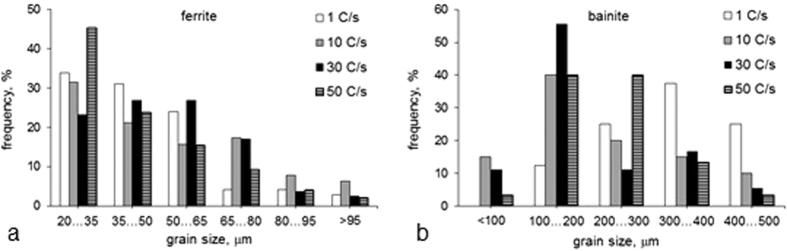
Grain size distributions for (**a**) ferrite and (**b**) bainite.

As can be seen from Fig. 1 and Table 1, with an increase in solidification rate, the average ferrite grain size varied insignificantly. However, the average bainite grain size and plate width decreased with an increase in solidification rate and showed a minimum at 30 Cs^−1^. These correspond to the variations in grain size distributions with solidification rate (Fig. 2): for the solidification rate of 30 Cs^−1^, the ferrite grain size distribution showed a minor shift towards larger grain sizes (observe a peak in the 50–65 μm range); in contrast, the bainite grain size distribution exhibited a considerable shift towards smaller grain sizes (observe a peak in the 100–200 μm range). The bainite phase fraction decreased with a decrease in solidification rate and showed a minimum at 30 Cs^−1^ (Table 1). These variations in grain structure could have resulted from a decrease in prior austenite grain size, due to a decrease in the secondary dendrite arm spacing and reduced grain growth rate with the increase in cooling rate. For example, in low carbon steels, a smaller prior austenite grain size has been observed to lead to a slower bainite transformation rate^18^ and a lower bainite fraction^19^. In addition, the austenite strength variation with solute segregation and particle precipitation could also affect the bainite transformation kinetics. A decrease in austenite strength increases the bainite plate thickness, as a result of faster propagation of the bainite-austenite interface in austenite that is free from strengthening agents (such as dislocation debris, solute atoms and precipitates); and a more chemically homogeneous austenite faster transforms to bainite, due to a more uniform nucleation of bainite^20^. Faster transformation and growth may lead to a larger bainite grain size and area fraction. These effects could take place here, at a 50 Cs^−1^ solidification rate. This requires further investigation.

**Figure 3:**
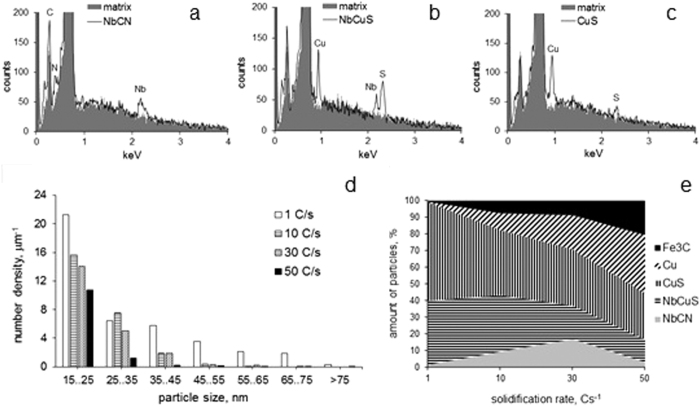
Typical EDS spectra of (**a**) NbCN, (**b**) NbCuS and (**c**) CuS particles of >15 nm size; effect of solidification rate on the (**d**) number density distributions and (**e**) chemistry variation of the >15 nm particles.

The SEM studies revealed 15–120 nm precipitates in both ferrite and bainite for all processing conditions (Fig. 1e–h). The particles were mainly comprised of NbCN, NbCuS, CuS, ε-Cu and Fe_3_C type (Fig. 3a–c). The particle parameters varied with solidification rate (Table 1, Fig. 3d,e). The average >15 nm particle number density and area fraction decreased with an increase in solidification rate. This can be explained by a decrease in time for particle growth with an increase in solidification rate. This is supported by the particle composition variations with solidification rate (Fig. 3e), where the amount of larger CuS and NbCuS particles decreased, while the percentage of smaller ε-Cu, and Fe_3_C particles increased, as the solidification rate increased. The relative amount of NbCN particles showed a maximum at a solidification rate of 30 Cs^−1^. Two scenarios could lead to this. In the first case, below 30 Cs^−1^ cooling rates (1 to 10 Cs^−1^, in this study) some Cu and S precipitated on NbCN core. However, the energy emissions from C and N were not detected because they were either masked by the heavier elements or the C and N atoms were not excited by the electron beam. In the second case, the segregation of Nb and S^21^ increased the local concentrations of these elements. This could promote NbS precipitation^22^ above the NbCN dissolution temperature and reduce the Nb content in the matrix available for NbCN precipitation. These require further investigation.

**Figure 4:**
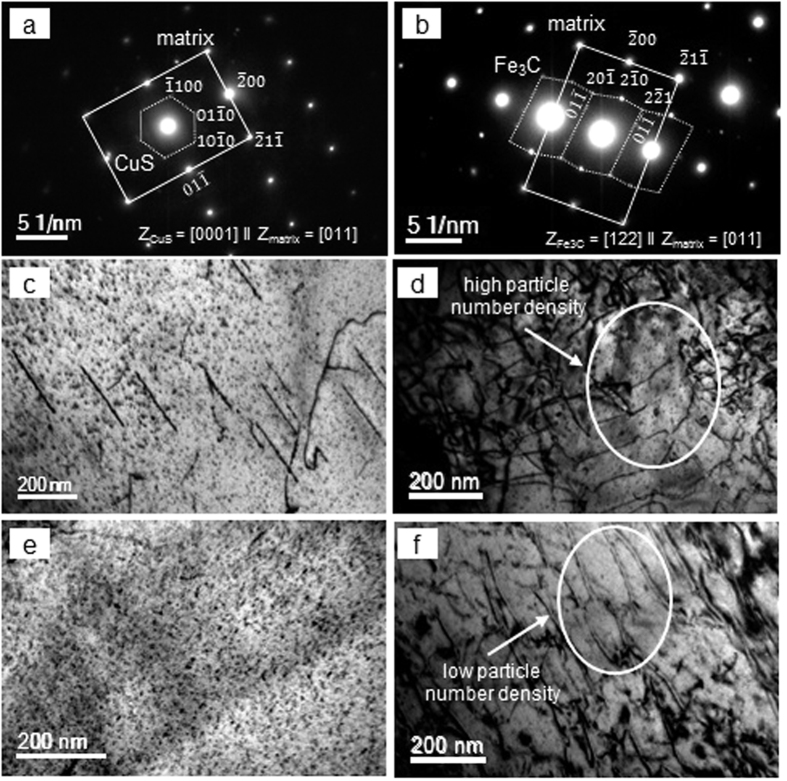
Typical selected area diffraction patterns of (**a**) CuS and (**b**) Fe_3_C particles; and TEM images of (**c**,**e**) ferrite (**d**,**f**) bainite for solidification rates of (**c**,**d**) 30 Cs^−1^, and (**e**,**f**) 50 Cs^−1^.

The TEM studies showed 4–13 nm precipitates in ferrite and bainite (Fig. 4). As the particles were too small for EDS, their nature was determined using the selected area diffraction technique. The particles in ferrite were predominantly Cu-rich (ε-Cu and CuS) and those in bainite were mainly Fe_3_C. A diffraction pattern showing the [0001]_CuS_ || [011]_matrix_ orientation relationship on Fig. 4a originated from the hexagonal crystal lattice, characteristic for CuS particles; and the measured 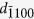 spacing of 0.321 nm was in good agreement with 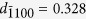 nm calculated based on the CuS lattice parameters a = 0.379 nm and c = 1.633 nm^23^. A diffraction pattern showing the [122]_Fe3C_ || [011]_matrix_ orientation relationship in Fig. 4b originated from the orthorhombic crystal lattice, characteristic for Fe_3_C particles^24^. The measured 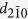 spacing of 0.287 nm was in good agreement with 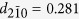 nm calculated based on the Fe_3_C lattice parameters a = 0.674 nm, b = 0.509 nm and c = 0.453 nm^25^. The average number density of <15 nm particle increased when the solidification rate increased (Table 1). However, the particle volume fraction at a cooling rate of 30 Cs^−1^ was more than 2 times higher than that at 50 Cs^−1^, due to a slightly larger average particle size at 30 Cs^−1^ (4–5 nm) compared to 50 Cs^−1^ (3–5 nm). It is clear that a higher solidification rate reduced the time available for the diffusion of atom, in particular carbon. This resulted in an increased number of nucleation sites of ≤15 nm particles, leading to decreased particle sizes with concurrent increased number density. The dislocation structure in ferrite was of a linear type (Fig. 4c). The average dislocation density in ferrite varied insignificantly with solidification rate and was measured to be about 0.3 × 10^14 ^m^−2^. The dislocation structure in bainite exhibited numerous knots and tangles (Fig. 4d,f). The average dislocation density in bainite was in the range of (1.4–2.7) × 10^14^ m^−2^, which is 6–8 times higher than that in ferrite. The dislocation density in bainite showed maximum during solidification at a cooling rate of 30 Cs^−1^, and this corresponds to the maximum number density of <15 nm particles in bainite. The dislocation pinning by these particles could have enhanced the generation of new dislocations^26^.

The average microhardness (and the bainite microhardness) showed a maximum for solidification at a cooling rate of 30 Cs^−1^. This corresponds to the minimum of bainite grain size and plate width, and maxima of the <15 nm particles number density and dislocation density in bainite. The average microhardness (264 HV) and maximum bainite microhardness (294 HV) obtained in this work are higher than those reported previously for a thin cast, microalloyed steel, of similar grade (about 260 HV in ref. 27). However, the Mn and Nb contents in our steel (0.5 and 0.059 wt. %) were lower than those in the steel reported in ref. 27, i.e 0.83 and 0.084 wt. %, while the cooling rates applied here were much lower than those expected in the reported work. Clearly, higher C and Mo contents in our steel increased its hardenability. With respect to processing, it is important to stress that our results were obtained via direct cooling without further heat treatment. In contrast, the work reported in reference^27^ has the steel held at 700 °C, for 4 min for age-hardening, which is a more expensive technology.

## Conclusion

In a low C, low Mn, NbTi-microalloyed steel, the continuous cooling at 1 °Cs^−1^ from 900 to 400 °C resulted in the formation of a dual-phase ferrite-bainite microstructure; however the phase balance varied with solidification rate, namely, the rate of cooling from 1600 to 900 °C. The solidification at 30 °Cs^−1^ resulted in about 50/50 phase balance, minimum bainite grain size and plate width, and maxima of the <15 nm particles number density and dislocation density in bainite. Such a combination of microstructural parameters led to the maximum strength observed following solidification at 30 °Cs^−1^.

## Additional Information

**How to cite this article**: Kostryzhev, A. G. *et al*. Effect of solidification rate on microstructure evolution in dual phase microalloyed steel. *Sci. Rep.* **6**, 35715; doi: 10.1038/srep35715 (2016).

## Figures and Tables

**Table 1:** Microstructural parameters.

		1 Cs^−1^		10 Cs^−1^		30 Cs^−1^		50 Cs^−1^	
parameter		ferrite	bainite	ferrite	bainite	ferrite	bainite	ferrite	bainite
grain size/plate width, μm		46 ± 19	312 ± 100 14 ± 5	51 ± 25	252 ± 120 13 ± 5	53 ± 23	202 ± 117 10 ± 4	42 ± 21	234 ± 84 11 ± 5
phase fraction, %		10	90	18	82	48	52	46	54
>15 nm particles (SEM)	size, nm	34	36	27	24	25	24	29	27
	ND*, μm^−2^	3	46	17	28	26	17	10	14
		42		26		21		12	
	area fraction	0.0021	0.0407	0.0106	0.0135	0.0140	0.0087	0.0029	0.0018
		0.0368		0.0130		0.0112		0.0023	
<15 nm particles (TEM)	size, nm	Not observed by TEM	5	Not studied		5	4	3	5
	ND, μm^−3^		3435			7148	6250	11944	2697
						6681		6950	
	volume fraction		0.0004			0.0008	0.0004	0.0003	0.0002
						0.0006		0.00025	
dislocation density, x10^14^ m^−2^			1.7 ± 0.4			0.3 ± 0.1	2.6 ± 0.1	0.3 ± 0.2	1.2 ± 0.1
hardness, HV		275 ± 6	240 ± 6	248 ± 10	252 ± 11	232 ± 12	294 ± 14	248 ± 8	243 ± 7
average hardness		244		251		264		245	

^*^ND is number density.
